# Examining the Efficacy of 38% Silver Diamine Fluoride in Halting Active Dental Caries Lesions Among Children: A Descriptive Study

**DOI:** 10.7759/cureus.56762

**Published:** 2024-03-23

**Authors:** Ayesha Saleem, Afshan Manzoor, Amara Nazir, Humara Iqbal, Muhammad A Khan, Zohaib Ahmed, Mustafa Sajid, Muhammad Kashif

**Affiliations:** 1 Operative Dentistry, Nishtar Institute of Dentistry, Multan, PAK; 2 Operative Dentistry, Bakhtawar Amin Dental College and Hospital, Multan, PAK; 3 Operative Dentistry, Bakhtawar Amin Medical and Dental College, Multan, PAK; 4 Operative Dentistry, Multan Medical and Dental College, Multan, PAK; 5 Oral Medicine, Bakhtawar Amin Medical and Dental College, Multan, PAK; 6 Periodontology, Shahida Islam Medical and Dental College, Lodhran, PAK; 7 Oral Pathology, Bakhtawar Amin Medical and Dental College, Multan, PAK

**Keywords:** remineralization, demineralization, topical fluoride, silver diamine fluoride, dental caries

## Abstract

Background/objectives: Dental caries, a prevalent issue among children, often leads to discomfort and potential complications. Utilizing caries-arresting treatments to slow down its progression offers a practical alternative. Previous research indicates that topical fluorides can deactivate and remineralize enamel caries. This study aims to evaluate the efficacy of 38% silver diamine fluoride (SDF) in halting active dental caries lesions in children.

Materials and methods: This descriptive case series was conducted at the Operative Dentistry Department of the Nishtar Institute of Dentistry in Multan, Pakistan. We enrolled 753 patients aged three to nine years, of both genders, each with at least one cavitated lesion graded 3-6 according to the International Caries Detection and Assessment System (ICDAS). SDF was directly applied to dried and isolated teeth using a micro brush and left to absorb for up to two minutes (adjusted based on the child's cooperation), and parents were instructed to ensure the child refrained from eating or drinking for an hour post-application. Baseline examinations were performed by consultant restorative dentists, and reexaminations were conducted after two to three weeks by a consultant unaware of the study.

Results: The study included children aged three to nine years, with a mean age of 6.02±1.35 years. The majority of patients (61.75%) were aged between three and six years. Among the 753 patients, 619 (82.20%) were male, and 134 (17.80%) were female, with a male-to-female ratio of 4.6:1. The efficacy of 38% SDF in arresting active dental caries lesions in children was observed in 720 (95.62%) patients.

Conclusion: This study demonstrates that 38% SDF is highly effective in halting active dental caries lesions in children.

## Introduction

Dental caries remains a prevalent issue in economically developing nations, affecting a substantial portion of children, ranging from 60% to 90% [1]. In Pakistan, oral health trends reflect alarming statistics, with dental caries emerging as the most widespread childhood ailment, surpassing conditions like asthma by fivefold and hay fever by sevenfold [2]. Left untreated, dental decay in preschoolers escalates the risk of future caries in both primary and permanent dentition, along with associated consequences such as pain, infections, costly emergency room visits, hospitalizations, extensive treatment requirements necessitating general anesthesia, hindered growth and development, and increased absenteeism from school [3]. The term "early childhood caries" (ECC) is now used to define decayed, missing, or restored teeth in the primary dentition of children [4]. ECC affects a significant portion of preschool populations today, with a disproportionate burden on disadvantaged families [5].

Caries-arresting treatments offer a practical approach to slowing down the progression of dental caries, thereby mitigating children's discomfort and averting associated consequences. Research has shown that topical fluorides can deactivate and remineralize enamel caries [6]. Various fluoride application methods, including public water supply fluoridation and professionally applied fluoride gels such as NaF, 1.23% APF, and SnF2, have been employed to prevent and arrest early childhood caries. Within this context, there is growing interest in silver diamine fluoride (SDF) for halting the advancement of cavities (caries lesions) post-onset. Topical application of SDF, a clear liquid, in milligram amounts, directly on the active lesion surface, effectively arrests the lesion [7]. The antimicrobial action of silver ions within SDF inhibits the growth of various oral bacteria and disrupts enzymes responsible for collagenous dentin breakdown. Additionally, fluoride promotes the formation of fluorapatite, which exhibits greater resistance to acidic degradation by *Streptococcus mutans* compared to normal hydroxyapatite tooth structure [8].

A study reported that out of 102 lesions, 100 were arrested at the first recall after three weeks, with all lesions arrested by the second recall after three months. No instances of pain or infection were recorded in teeth treated with SDF. Parental feedback regarding ease of application, taste, and aesthetics was positive [9]. Given the unique combination of dietary patterns, oral hygiene practices, and the relatively lower level of parental education within the local context, there has been a notable absence of research pertaining to the utilization of SDF for the arrest of dental caries lesions in Pakistan. Therefore, the primary aim of this study was to evaluate the efficacy of 38% SDF in halting the progression of active dental caries lesions among children within this distinctive demographic setting.

## Materials and methods

Study settings and participants

This descriptive case series study was conducted in the Operative Dentistry Department of the Nishtar Institute of Dentistry in Multan, Pakistan. Approval for the said study was obtained from the Institutional Research Board (IRB) of the Nishtar Institute of Dentistry (approval number: CPSP/REU/DSG-2018-102-2428). A sample size of 753 patients was determined based on an anticipated SDF efficacy of 98%, a confidence level of 95%, a significance value (α) of 5%, and a margin of error of 1%. Non-probability consecutive sampling was employed to recruit children aged three to nine years of both genders, each with at least one cavitated lesion, graded 3-6 according to the International Caries Detection and Assessment System (ICDAS). Exclusion criteria encompassed children with pulpitis, tooth mobility, hereditary developmental defects of teeth, and known allergies or sensitivities to dental materials, including SDF.

Clinical procedure

Informed consent was obtained from all enrolled patients presenting to the Operative Dentistry Department. Demographic details including age, gender, location of caries (right or left side of the arch, upper or lower arch), tooth number, and their respective ICDAS scores were recorded. ICDAS scores were recorded according to the following classifications of enamel caries: Code 0: indicates a sound tooth with no evidence of caries, showing either no change or questionable change in enamel translucency after air-drying for five seconds; Code 1: represents the first visual change observed in enamel, noticeable only after air-drying for five seconds; Code 2: denotes a distinct change observed in enamel; Code 3: signifies the localized breakdown of enamel without gross clinical signs indicating progression to dentin; Code 4: indicates a shadow observed from dentin; Code 5: represents a distinct carious cavity with visible dentin; and Code 6: denotes an extensive distinct carious cavity with visible dentin.

Potential effect modifiers such as bottle feeding habits (particularly night-time), tooth brushing frequency (less than twice daily), and soft drink consumption (with or without straw) were also documented. SDF was applied directly to the dried and isolated teeth on the lesion using a micro brush and allowed to absorb for up to two minutes, with parents instructed to ensure the child refrained from eating or drinking for an hour post-application. Proper isolation and drying of the tooth were emphasized to optimize SDF's effectiveness in arresting the lesion. Baseline examinations were conducted by a consultant with at least three years of post-fellowship experience, and reexaminations were performed after two to three weeks by a consultant unaware of the study, assessing for the arrest of caries lesions or improvement by at least one ICDAS score. SDF treatment was deemed ineffective if the ICDAS criteria increased by one number after the three-week follow-up. All data were meticulously recorded on a standardized form.

Data analysis

Data analysis was performed using IBM SPSS Statistics for Windows, Version 21.0 (Released 2012; IBM Corp., Armonk, New York, United States). Quantitative variables such as age were presented as mean and standard deviation, while qualitative data including gender, tooth number, ICDAS score, night-time bottle feeding, soft drink consumption, and SDF effectiveness were presented as frequencies and percentages. Stratification based on age groups, gender, tooth brushing frequency, bottle feeding habits, and soft drink consumption was conducted to evaluate their impact on effectiveness. Post-stratification chi-squared tests were applied, with p-values ≤0.05 considered significant.

## Results

Table 1 provides a summary of key variables studied, including the mean age of participants, distribution across age groups, gender composition, tooth brushing habits, and ICDAS scores before and after intervention. The average age of participants was 6.02 years, with a standard deviation of 1.35 years. Most participants fell within the three- to six-year age group, comprising n=465 (61.75%) of the total sample, while n=288 (38.25%) were aged seven to nine years. Gender distribution showed a predominance of males, constituting n=619 (82.2%) of the sample, with females comprising n=134 (17.8%). Regarding tooth brushing frequency, n=265 (35.19%) reported brushing twice a day, while n=488 (64.81%) did not. The mean ICDAS score before intervention was 4.80±1.26, decreasing to 1.92±0.83 after intervention, indicating a significant improvement in dental caries status following the implemented treatment.

**Table 1:** General characteristics of study subjects ICDAS: International Caries Detection and Assessment System

Variable		Value
Mean age (years)		6.02±1.35 years
Age groups	3-6 years	465 (61.75%)
	7-9 years	288 (38.25%)
Gender	Male	619 (82.2%)
	Female	134 (17.8%)
Tooth brushing twice a day	Yes	265 (35.19%)
	No	488 (64.81%)
Mean ICDAS score	Before	4.80±1.26
	After	1.92±0.83

**Figure 1:**
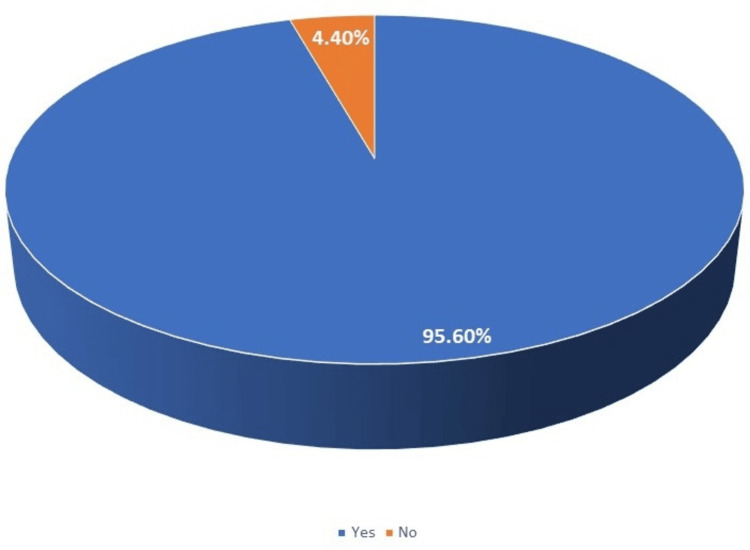
Effectiveness of 38% silver diamine fluoride in arresting active dental caries lesions in children (n=753)

In our study, the effectiveness of 38% SDF in arresting active dental caries lesions in children was found in n=720 (95.62%) patients (Figure 1).

Table 2 presents the effectiveness of the intervention across different demographic groups, along with associated p-values. Effectiveness is measured as the percentage of individuals for whom the intervention was successful (labeled "Yes") versus those for whom it was not (labeled "No"). The groups are stratified based on age (3-6 years and 7-9 years), gender (male and female), and tooth brushing frequency (twice a day and not twice a day). For each group, the table indicates the number and percentage of individuals in each effectiveness category ("Yes" or "No"). Additionally, p-values are provided to assess the statistical significance of differences in effectiveness between groups. The results suggest that there are no statistically significant differences in effectiveness based on age (p=0.454), gender (p=0.055), or tooth brushing frequency (p=0.605).

**Table 2:** Comparison of the effectiveness of 38% silver diamine fluoride with respect to age, gender, and tooth brushing habit

Groups		Effectiveness		P-value
		Yes	No	
Age (years)	3-6	446 (95.91%)	19 (4.09%)	0.454
	7-9	274 (95.14%)	14 (4.86%)	
Gender	Male	596 (96.28%)	23 (3.72%)	0.055
	Female	124 (92.54%)	10 (7.46%)	
Tooth brushing twice a day	Yes	252 (95.09%)	13 (4.91%)	0.605
	No	468 (95.90%)	20 (4.10%)	

## Discussion

Dental caries prevention and management are pivotal in pediatric dental care [10]. Preventive measures for ECC are not only cost-effective but also crucial in averting the need for expensive emergency room visits or extensive restorative treatments [11,12]. Although various evidence-based approaches for caries prevention exist, their implementation often requires substantial financial resources and access to oral health facilities and professionals. Effective ECC preventive measures include the application of fluoride varnish, such as 5% sodium fluoride (NaF), and the use of fluoridated toothpaste [13-16]. Atraumatic restorative treatment (ART) is recommended for managing cavitated ECC, offering a painless and cost-effective alternative to conventional treatment, particularly in settings with limited resources; however, its efficacy is limited by a high failure rate [17]. SDF has garnered increasing interest as an alternative treatment for caries prevention and arrest [18]. In vitro studies have demonstrated SDF's ability to increase biofilm pH, reduce dentin demineralization, and exhibit antimicrobial properties against cariogenic bacteria [19]. However, SDF-treated teeth may develop black stains due to silver phosphate precipitation [20]. Ex vivo and in vivo studies on extracted teeth from children receiving semiannual SDF applications have shown promising results in arresting lesions and enhancing fluoride uptake compared to fluoride varnish and acidulated phosphate fluoride gel [21,22]. This study aims to evaluate the effectiveness of 38% SDF in halting active dental caries lesions in children. Our findings demonstrate a high efficacy rate of 95.62% in arresting active caries lesions with 38% SDF treatment. Clinical trials have reported significantly fewer new caries lesions and a higher proportion of inactive caries surfaces in children treated with 38% SDF compared to controls [23]. Moreover, semiannual application of 38% SDF has shown superior caries arrest rates compared to annual application or alternative treatments such as glass ionomer cement (GIC) [24]. Although one study suggested that SDF was less effective than ART sealants, discrepancies in trial design and follow-up may have influenced the outcomes [25]. Another study comparing the effectiveness of 30% SDF versus fluoride varnish found that multiple SDF applications led to significantly higher caries arrest rates than fluoride varnish, albeit with varying efficacy over time [26].

Several limitations need to be acknowledged in this study. Firstly, the research was conducted within a specific timeframe and setting, limiting the generalizability of the findings to other populations or contexts. Secondly, the study design, being a descriptive case series, may not provide strong evidence of causality, and there could be confounding variables influencing the observed outcomes. Furthermore, the assessment of treatment effectiveness relied on subjective measures such as clinical evaluations, which could introduce bias or variability in interpretation. Moreover, the follow-up period in this study might have been relatively short to capture long-term outcomes or potential adverse effects of the intervention. Finally, the reliance on a single treatment modality, 38% SDF, limits the comparison with other interventions or treatment combinations that could have provided a more comprehensive understanding of the efficacy and potential drawbacks. These limitations highlight the need for further research with robust study designs, longer follow-up periods, and consideration of various treatment approaches to better inform clinical practice and policy decisions.

## Conclusions

The findings of this study underscore the remarkable efficacy of 38% SDF in halting active dental caries lesions among children. With an impressive success rate, the application of SDF presents a promising intervention for managing dental caries in pediatric patients. Beyond merely arresting the progression of caries, the use of SDF offers a non-invasive, cost-effective solution that could potentially alleviate the burden of dental decay among young populations. As such, integrating 38% SDF into routine dental care protocols could significantly contribute to improving oral health outcomes and reducing the need for invasive interventions in pediatric dentistry. However, long-term follow-up studies are warranted to assess the durability of SDF treatment effects and its impact on preventing future caries development, ensuring its sustained effectiveness in pediatric dental practice.
